# The Significance of Rhizobacteria for Strawberry Cultivation in Tropical Area: A Review

**DOI:** 10.21315/tlsr2025.36.2.15

**Published:** 2025-07-31

**Authors:** Febby Fitriyani, Etty Pratiwi, Masako Akutsu, Reginawanti Hindersah, Syariful Mubarok

**Affiliations:** 1 Masters student of Soil Science, Faculty of Agriculture, Universitas Padjadjaran, Jalan Raya Bandung Sumedang KM 21, Jatinangor 45363, Indonesia; 2 Research Center for Food Crops, National Research and Innovation Agency, Indonesia; 3 Faculty of Agriculture, Shinshu University, 8304, Minami-minowa, Kami-ina, Nagano 399-4598, Japan; 4 Department of Soil Science and Land Resources, Faculty of Agriculture, Universitas Padjadjaran, Jalan Raya Bandung Sumedang KM 21, Jatinangor 45363, Indonesia; 5 Department of Agronomy, Faculty of Agriculture, Universitas Padjadjaran, Jalan Raya Bandung Sumedang KM 21, Jatinangor 45363, Indonesia

**Keywords:** Biofertiliser, Rhizobacteria, Review, Strawberry, Tropical

## Abstract

In tropical regions, high temperatures and low nitrogen (N) and phosphorus (P) in soil limit plant performance and fruit production. The soil-beneficial microbes, including rhizobacteria, have the potential to overcome the nutrient problems in the soil. Rhizobacteria fix the dinitrogen, solubilise the P and potassium (K), and produce hormones and other metabolites to stimulate plant development and resistance against environmental challenges like inadequate soil fertility, heavy metal concentrations or drought. Bacterial genera that occur for promoting growth is *Bacillus* sp., *Azospirillum* sp., *Azotobacter* sp, *Pantoea* sp. and *Pseudomonas* sp. Despite the prominent role of rhizobacteria in agriculture and the economic value of strawberries, the potential use of rhizobacteria as a biofertiliser in strawberry cultivation in tropical areas is rarely discussed and reviewed. The information obtained from publications from 2014–2023 by using the keywords of Plant Growth Promoting Rhizobacteria (PGPR), tropics, biofertiliser, N fixation, P and K solubilisation, P mineralisation, phytohormones and strawberry is organised according to the rhizobacteria, mechanisms by which they boost plant growth, and research location in tropical area. This review focuses on evaluating (i) the mechanism of rhizobacteria to increase plant growth, (ii) the role of rhizobacteria on strawberry growth, yield and quality and (iii) the impact of rhizobacteria on biotic and abiotic stress alleviation.

**Highlights**

- Rhizobacteria enhance strawberry growth, yield and quality: The presence of beneficial rhizobacteria (e.g., *Bacillus* sp., *Azospirillum* sp., *Azotobacter* sp., *Pantoea* sp. and *Pseudomonas* sp.) has been demonstrated to enhance nutrient availability and promote plant growth by nitrogen fixing, solubilising phosphorus and potassium, and producing phytohormones and exopolysaccharides (EPS).
- Rhizobacteria enhance plant resilience: The rhizobacteria play a pivotal role in mitigating abiotic stresses and against pathogens as biocontrol agents that are prevalent in tropical climates.
- Potential for sustainable strawberry cultivation in tropical regions: The rhizobacteria-based biofertilisers offers a sustainable alternative to chemical fertilisers, thereby reducing environmental impact while maintaining high agricultural productivity, emphasising their role in sustainable tropical agriculture.

## INTRODUCTION

Strawberry (*Fragaria × ananassa*, Duch.) is a hybrid of Rosaceae family plants, *Fragaria chiloensis* and *Fragaria virginian* (de Moura *et al.* 2022). It holds substantial economic value across various industries (Simpson 2018). Major producers such as China, the United States and Mexico collectively contribute half of the global supply, which exceeded 9 million tons in 2019 (FAO). Cultivation spans tropical, subtropical and temperate zones, with tropical cultivation typically at high altitudes in mountainous areas (de Andrade *et al.* 2019).

Due to high drought, temperature and soil-borne diseases, challenges persist in tropical cultivation (Khammayom *et al.* 2022). Intensive weathering processes in tropical regions result in low soil acidity, organic carbon and limited nitrogen and phosphorus availability (Piamonte *et al.* 2014). High precipitation can lead to nutrient leaching due to organic matter degradation, which further compounds these challenges (Quan *et al.* 2022).

Open-field cultivation is commonly practiced, but soil-borne diseases pose a significant threat. Chemical fertilisers are extensively used to augment nutrient deficiencies, increasing yield (Maurya *et al.* 2017). *Phytophthora* and *Verticillium* are essential pathogens that cause rotting and wilting diseases in strawberries (Fan *et al.* 2018). Inoculating with plant growth-promoting rhizobacteria (PGPR) has shown promise in enhancing growth and stress tolerance, offering eco-friendly and cost-effective solutions to increase strawberry yield (Naamala & Smith 2020).

**Figure 1:**
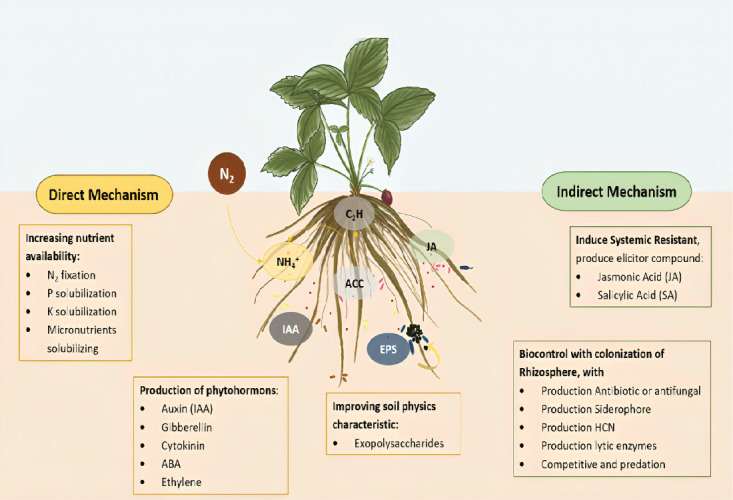
Rhizobacteria mechanism as direct and indirect to increase plant development.

Rhizobacteria application as biostimulants, biofertilisers or biocontrol agents has surged, recognised for their economic benefits, enhanced yields and environmental sustainability (Koskey *et al.* 2021). The bacteria directly influence plant growth by providing nutrients through nitrogen fixation and phosphate and potassium solubilisation, phytohormones and disease protection alongside indirect mechanisms involving the production of volatile compounds, siderophores, exopolysaccharides and antibiotics (Fig. 1). Rhizobacteria are crucial in inducing systemic resistance, reducing disease intensity and increasing plant development against stressful conditions, significantly contributing to plant resilience (Naamala & Smith 2020).

Widely used genera of rhizobacteria are Nitrogen fixer bacteria (NFB) such as *Azotobacter, Azospirillum, Bradyrhizobium* and *Rhizobium*; Phosphate solubilising bacteria (PSB) such as *Bacillus* sp. and *Pseudomonas* sp. and Potassium solubilising bacteria (KSB), *Bacillus mucilaginous* (Azizoglu 2019). Numerous other genera have been shown to promote plant growth and produce phytohormones, such as auxin, gibberellin and cytokinin (Chebotar *et al.* 2022). Certain *Bacillus* species have been reported to enhance the percentage of significant macroaggregates, improving the soil structure and root development through exopolysaccharides (EPS) production (Vikram *et al.* 2022; Costa *et al.* 2018).

Balanced nutrient management, incorporating chemical, organic and microbial-based fertilisers, is advocated for sustainable growth (He & Dijkstra 2014). Rhizobacteria play a crucial role in improving soil and fruit quality while enhancing plant resilience to various stressors (Hindersah *et al.* 2019; Redondo-Gómez *et al.* 2022). This review reported the potential role of rhizobacteria in promoting strawberry growth and productivity in tropical regions, emphasising their role in the soil and plant health.

## RHIZOBACTERIA MECHANISM FOR INCREASING PLANT GROWTH

In tropical soils, where nitrogen, phosphorus and potassium availability is low, rhizobacteria enhance the efficient use of chemical fertilisers by reducing fertiliser doses (de Andrade *et al.* 2019). According to Le *et al.* (2019), they contribute to nitrogen fixation, phosphate and potassium solubilisation, and stress tolerance promotion. Rhizobacteria synthesise organic acids and enzymes, which solubilise and mineralise P, aiding in heavy metal stress tolerance (Ducousso-Détrez *et al.* 2022). Additionally, they enhance plant K availability by gluconic and oxalic acid, which solubilise insoluble K in soil (Olaniyan *et al.* 2022).

The rhizosphere is inhabited by NFB that stimulate plant development by converting molecular N_2_ to ammonia gas (NH_3_), catalysed by nitrogenase, an oxygen-sensitive enzyme complex (Le *et al.* 2019). The N fixation reaction is stated elsewhere as the N reduction process:


(1) $${\text{N}}_{2}+8\;{\text{H}}^{+}+8\;{\text{e}}^{-}\to 2{\text{NH}}_{3}+{\text{H}}_{2}$$

The N fixation requires 16 adenosine triphosphate molecules to convert one molecule of N_2_ to NH. Under nitrogen fixation, PII signal transduction protein interacts with the RnfC gene, which controls electron flow to control electron transfer to nitrogenase (Batista & Dixon 2019).

The available form of N readily uptake by roots are NH_4_^+^ and NO_3_^−^. In the non-symbiotic NFB, NH_3_ is released from the bacteria into the soil by diffusion (Haskett *et al.* 2022). Dissolved NH_3_ in soil solution exists in chemical equilibrium with ammonium cations, the NH_4_^+^ (du Preez & Burger 1988). The NH_4_^+^ in soil solution will be converted to NO_3_^−^ by 2-step enzymatic nitrification involving ammonia oxidation to nitrite by chemolithotrophic ammonia-oxidizers and nitrite oxidation to nitrate by nitrite-oxidizers (Levy-Booth *et al.* 2014):


(2.) $$2{{\text{NH}}_{4}}^{+}+3{\text{O}}_{2}\to 2{{\text{NO}}_{2}}^{-}+4{\text{H}}^{+}+2{\text{H}}_{2}\text{O}$$

(3) $$2{{\text{NO}}_{2}}^{-}+{\text{O}}_{2}\to 2{{\text{NO}}_{3}}^{-}+\text{energy}$$

In contrast, another research ensures that fixed N in nonsymbiotic NFB is assimilated into bacterial biomass and not excreted into the soil (Batista & Dixon 2019). Meanwhile, symbiotic NFB provides plants with asparagine, an amino acid synthesised in the plant cell by using glutamate released by rhizobia living in the nodule (Schwember *et al.* 2019). Specific mechanisms to increase the availability of N in the soil by nonsymbiotic NFB have not been explicitly stated. However, many studies have shown the impact of this bacteria on the available N increment in soil (dos Santos Cordeiro & Echer 2019; Haerani *et al.* 2021; Mendes-Santos *et al.* 2017).

The rhizobacteria are involved in the P and K cycle in soil. They produce the organic acid to release phosphate from inorganic P (Pi) of Ca, Fe and Al to become available for plants (Ducousso-Détrez *et al.* 2022). The organic acid also solubilises K minerals, including micas, muscovite, feldspar, biotite, illite and orthoclase (Olaniyan *et al*. 2022). The well-known short-chain organic acids (C2–C6 acids) synthesised by rhizobacteria to solubilise the P and K are lactic, citric, acetic and succinic acids (Naraian & Kumari 2017; Zhao *et al.* 2024). They are essential intermediate metabolites in bacterial cells and are commonly produced from sugar via the microbial Tricarboxylic acid cycle and fermentation (Sun *et al.* 2020).

The low-molecular-weight organic acids solubilise the fixed inorganic P (Pi) by lowering the soil pH, chelating cations and competing with ortophosphate (PO_4_^−^) for adsorption sites in the soil (Saeed *et al.* 2021). The organic acid can solubilise the Pi on soil colloids as chelators of cations such as Fe, Al^3+^, and Ca^2+^ and compete for P adsorption sites in soil (Menezes-Blackburn *et al.* 2016). They also form a complex metal cation chelation with base cations on soil such as K^+^, Mg^2+^, and Ca^2+^ as readily for plant uptakes (Vega *et al.* 2022). During the process, organic acid has a mechanism for soil acidification as the metal becomes soluble and is released into the soil solution (Achor *et al.* 2020).

Organic P (OP) in soil accounts for 42% of the P pool (Menezes-Blackburn *et al.* 2018). OPs contain phosphate groups or P bonded to carbon (C) groups, including orthophosphate monoesters, orthophosphate diesters and phosphonates (Cade-Menun 2017) originating from microbial cells and plant and animal debris. Bacterial phosphatases catalyse the OP mineralisation to available inorganic P (Pi) based on the type of OP substrates (Park *et al.* 2022). The Pi enters the bacterial cytoplasm via a transporter for cell metabolism, and part of the Pi is adsorbed by roots. Major (N, P and K) elements available for plants are essential in reducing the impact of stress conditions. Many studies have demonstrated that essential nutrients such as N and P are related to the mitigation of abiotic stress in plants and the reduction of the effect of drought stress (He & Dijkstra 2014).

The detailed biosynthesis pathway of various phytohormones by individual bacteria is limited. Generally, the rhizobacteria, including *Azospirillum, Bacillus, Pseudomonas* and *Rhizobium*, synthesise the indole acetic acid (auxin) via the tryptophan-dependent pathways (Tang *et al.* 2023). Naturally occurring CKs are adenine derivatives; the microbes synthesise the CK via De novo CK and tRNA-dependent CK biosynthesis pathway (Frébortová & Frébort 2021). Gibberellins (GA) are phytohormones characterised by a complex diterpenoid structure. The biosynthetic pathways of GA in plants and fungi have been elucidated, although knowledge on GA biosynthesis in bacteria remains limited. Symbiotic nitrogen-fixing *Bradyrhizobium japonicum* and *Sinorhizobium fredii* have a putative GA biosynthetic operon/gene cluster for encoding the enzymes to produce GA9 (Nett *et al.* 2017).

The challenge of growing strawberries in the field is the soil’s physical quality. Tropical soil generally has poor physical properties due to low organic C and high clay content, which causes restrained structure and poor infiltration (Zhong *et al.* 2018). EPS produced by rhizobacteria can adhering soil particles by forming aggregates around the roots (Costa *et al*. 2018; Sivapriya 2018). They also can improve water holding capacity and reduce proline accumulation and antioxidant enzyme activity as tolerant mechanism for drought stress conditions (Sandhya & Ali 2015; Naseem *et al.* 2024). EPS of rhizobacteria such as *Pseudomonas bathysetes* secrete EPS to improve porosity, bulk density, and soil aggregate stability to improve micro aggregation (Dar *et al.* 2021; Olagoke *et al.* 2022). Application of some *Bacillus* species increases the percentage of large macro aggregates of > 2 mm (Vikram *et al.* 2022). This improves the soil structure by increasing infiltration and providing better porosity for root development (Sharma *et al.* 2018).

EPS produced by rhizobacteria enables soil particles to form aggregates around the roots, enhances water-holding capacity and facilitates the plant’s uptake of nutrients and minerals. Rhizobacteria produce EPS as a protective mechanism to shield themselves from abiotic stressors like extreme temperatures, metal concentration, drought and biotic threats (Carezzano *et al.* 2023). The mechanism of EPS is to protect bacteria and plant hosts from abiotic stress by maintaining and regulation nutrient uptakes and absorption as chelator ions (Paul *et al.* 2024). Moreover, EPS as a biopolymer is involved in metal-binding to facilitate heavy metal biosorption (Dhanya *et al.* 2021). The EPS acts as a chelator by forming COO^−^ (carboxyl group) and OH^−^ (hydroxyl ion) to bind cations, including metals, on soils. The EPS of *Azotobacter* sp. binds Cd and Cr in the contaminated soil (Rasulov *et al.* 2015) to reduce their availability. However, the excretion of EPS is also a mechanism for maintaining the balance of C/N ratio when the nitrogen in environment is sufficient (Qian *et al.* 2022). Therefore, EPS as sorption and retention mechanism may thus affect the mobility and fate of microbially-derived carbon (C), nitrogen (N) and phosphorus (P) in soils (Zhang *et al.* 2021).

### Role of Rhizobacteria Strawberry Growth, Yield and Quality

In tropical soils, where nitrogen, phosphorus and potassium are often limited, rhizobacteria are vital in fixing nitrogen and solubilising phosphorus and potassium (Hanyabui *et al.* 2020). However, soil-borne pathogens pose a challenge, leading to damping-off, root rot and vascular wilts exacerbated by continuous cropping (Arora *et al*. 2022). Soilless substrates, typically composed of organic matter and inorganic materials, offer a pathogen-free alternative but lack sufficient nutrients, necessitating fertiliser applications (Hindersah *et al.* 2022). Rhizobacteria application is feasible in soil-based and soilless cultivation systems (Table 1).

Rhizobacteria function as biofertilisers, providing essential nutrients through nutrient-solubilising mechanisms (Hindersah *et al.* 2022). They enhance strawberry development and yield by improving the nutrient supply and phytohormone production (Meena 2018; Liu *et al.* 2022). These microorganisms, including *Azotobacter* sp. and Burkholderia sp, positively impact strawberry development and yield by increasing nutrient content (Kumar *et al.* 2020). Various rhizobacteria, such as *Azospirillum brasilense* and *Bacillus megaterium*, stimulate plant growth by solubilising phosphorus and potassium while synthesising indole-3-acetic acid (da Silva *et al.* 2022). They can significantly reduce inorganic fertiliser consumption, with studies reporting biomass increases and root parameter enhancements following their application (Hindersah *et al.* 2021).

Table 2 shows the impact of rhizobacteria on strawberry development and productivity in soilless substrates and Table 3 shows the impact of rhizobacteria on the quality of strawberries.

Rhizobacteria boost growth and yield and enhance fruit quality through increased nutrient availability and ripening regulation (Negi *et al.* 2021). Potassium, crucial for fruit quality and stress responses, influences pathogen resistance and fruit ripening (Singh *et al.* 2020). Reduced nitrogen, phosphorus, potassium and biofertiliser applications have significantly improved fruit quality and yield (Nisarga *et al.* 2020). Various bacteria strains from *Bacillus* sp. and *Pseudomonas* sp. are employed as biofertilisers to enhance fruit quality (Nam *et al.* 2023).

## IMPACT OF RHIZOBACTERIA ON BIOTIC AND ABIOTIC STRESS

### Drought Stress Alleviation

**Figure 2:**
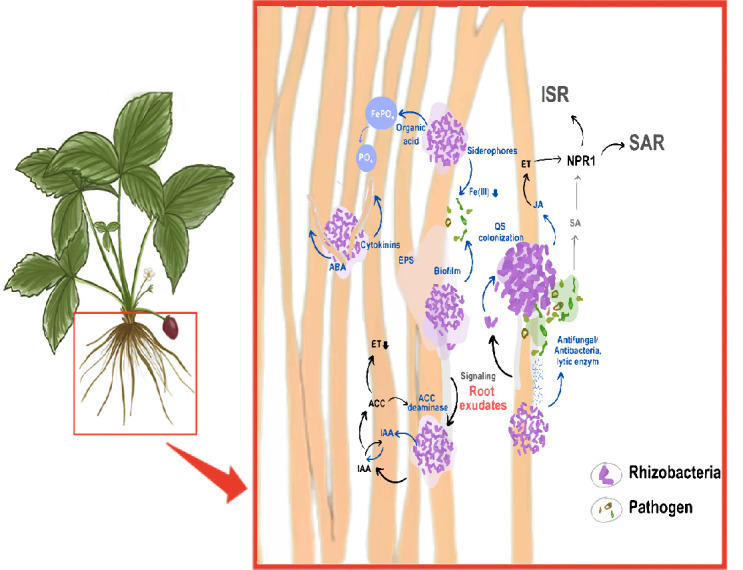
Mechanisms of rhizobacteria on biotic and abiotic stress through ISR and ASR.

Strawberries are highly vulnerable to drought, causing physiological stress and reducing fruit production and quality (Murthy & Pramanick 2014). In tropical open field areas, drought can lead to yield loss of up to 17% (Kumar *et al.* 2022). Nitrogen, phosphorus and potassium are essential nutrients that help plants adapt to abiotic stress, improve soil fertility and increase plant tolerance to environmental stresses (He & Dijkstra 2014). The shape and physiological functions of strawberry fruits are affected by water deficiency because of their depthless root systems, broad leaf areas and high fruit water content (Adak *et al.* 2018; Mozafari *et al.* 2018). Induced systemic resistance (ISR) and acquired systemic resistance (ASR) play crucial roles in biotic and abiotic resistance, respectively (Fig. 2). ISR enhances defense against pathogens, while ASR helps plants withstand environmental stresses vital for survival and productivity in challenging conditions.

Jasmonic acid (JA) is synthesised to respond to plant damage caused by pest attacks or pathogen colonisation. JA also induces a signalling pathway that leads to the production of ethylene hormones (Ma *et al.* 2020). The interaction between JA and ethylene activates defense-related genes, which express plant resistance and ISR (Ravanbakhsh *et al.* 2018). When a pathogen invades the plant, it also recognises the signalling of salicylic acid (SA) biosynthesis (Mishra *et al.* 2024). The accumulation of SA leads to the expression of pathogen-related genes, which secrete metabolites to enhance resistance to pathogens, resulting in systemic acquired resistance (Kim & Lim 2023).

In response to drought stress, organisms accumulate osmolytes, including betaines, sugars, polyols, polyamines and proline (Ashraf & Foolad 2007; Giri 2011). In strawberries, drought reduces chlorophyll and carotenoid pigments and relative water content while enhancing antioxidant enzyme activity, osmolyte accumulation and oxidative markers (Zahedi *et al.* 2023). Several genera of bacteria elevate osmolyte concentration, mitigate oxidative damage and bolster drought tolerance (Kour *et al.* 2022). These bacteria can break down ethylene precursors by secreting 1-aminocyclopropane-1-carboxylate (ACC) deaminase, thus enhancing plant resilience (Brunetti *et al.* 2021).

For instance, *Azospirillum* sp. enhances root development in tomatoes and xylem development in *Brassica competes* under drought conditions (Molina-Favero *et al.* 2008; Timmusk *et al.* 2014). Inoculation of several strains of *Bacillus* sp. and *Pantoea* sp. strains increases ACC deaminase production, auxin synthesis and phosphate-solubilising ability in strawberries (Paliwoda *et al.* 2022). However, comprehensive studies on rhizobacteria’s role in alleviating ‘strawberries’ drought stress are ongoing.

### Rhizobacterias as Biocontrol Agents

Wet tropical climates are marked by abundant rainfall and high humidity, and fungal and bacterial pathogens thrive, presenting substantial risks to strawberry plants (Morkeliūnė *et al*. 2021). Disease in such conditions includes black root rot, stem rot, crown rot and powdery mildew with key pathogens including *Verticillium* sp., *Botrytis cinerea*, *Colletotrichum* sp. and *Phytophthora* sp. (Drobek *et al.* 2021; Abdel-Gaied *et al.* 2022). Chemical pesticides can be effective but raise environmental and health concerns. Therefore, non-pathogenic rhizobacteria show promise as a substitute for chemical pesticides.

Many rhizobacteria are effective biocontrol agents (BCAs) for crop protection. Despite colonising the rhizosphere, they alleviate the detrimental effects by controlling plant disease and triggering immune responses (Abd-El-Kareem *et al.* 2021). Directly, rhizobacteria combat soil-borne pathogens by synthesising antimicrobial compounds such as antibiotics, siderophores, bacteriocin and volatile compounds (Raaijmakers *et al.* 2002; Subramanian & Smith 2015; Ryu *et al.* 2005; Vlassi *et al.* 2020). Rhizobacteria such as *B. Amyloliquefaciens* inhibit the mycelial growth of *F. solani* through the secretion of lipopeptide substances (Yang *et al.* 2024). Rhizobacteria also enhance plant health by providing nutrients and promoting better root growth via bacterial exopolysaccharide (EPS) and phytohormone production. Additionally, they produce antimicrobial substances, competing for nutrients and space (Grover *et al*. 2021). Indirectly, rhizobacteria can synthesise microbes-to-plant signals such as Lipo-chitooligosaccharides (LCOs) and microbes-to-pathogen signals as volatile organic compounds (VOCs) to provoke and induce systemic resistance (Jiao *et al.* 2021). *Azotobacter chroococcum, Azospirillum brasilense* and *Pseudomonas brassicacearum* induces phytohormonal signalling (jasmonic acid and ethylene) of plants due to population of *Tetranychus urticae* by regulating the content of phenolics, flavonoids and anthocyanins (Hosseini *et al.* 2022). Some studies have investigated the biocontrol mechanisms of rhizobacteria, encompassing direct and indirect approaches (Table 4).

## CONCLUSION

Beneficial rhizobacteria are essential for enhancing strawberry growth and production, especially in soilless-substrate cultivation in tropical regions. They directly contribute to plant growth by fixing nitrogen, solubilising phosphorus and potassium, producing phytohormones, and synthesising EPS. They also indirectly alleviate harsh conditions like drought and diseases, improving strawberry yield and quality. With low nutrient availability in tropical soil, N_2_-fixing, phosphate-solubilising and potassium-solubilising bacteria become crucial for promoting plant development.

Rhizobacteria function as biocontrol agents, managing pathogen growth in tropical regions with high disease intensity. Their diverse roles contribute to improving strawberry cultivation practices while decreasing dependence on chemical fertilisers, thus fostering environmental sustainability.

This literature review highlights the potential of various rhizobacteria to increase strawberry production and minimise chemical fertiliser usage in tropical climates. Their application proves significant in field-based and soilless cultivation, aiding strawberries to thrive despite drought and abiotic factors. Rhizobacteria offer promising prospects for sustainable strawberry cultivation in tropical regions.

## Figures and Tables

**Table 1:** Responses of strawberries on rhizobacteria inoculation in soil-based substrates and field soil.

Mechanisms	Impact	Species of rhizobacteria	References
Increase in nutrient availability	Improve plant development, productivity and nutritional content under saline stress.	*Alcaligenes* 637Ca and *Pseudomonas* 53/6	(Arikan *et al.* 2020)
	Increase the number of roots, root activity and improve nutrient uptake.	*Piriformospora indica*	(Liu *et al.* 2022)
	Induce earliness in flowering, fruiting and increase yield, quality fruits by substitute 25% of chemical fertiliser.	*Azotobacter* sp., *Azospirillum* sp.	(Kumar *et al.* 2019)
	Increase plant growth, yield and total antioxidant content.	*B. amylolequifaciens* BChi1 and *Paraburkholderia fungorum* BRRh	(Rahman *et al.* 2018)
Phytohormone production	Increase proline and protein levels in the leaves and improve chlorophyll content and leaf area.	*Alcaligenes* 637Ca and *Pseudomonas* 53/6	(Arikan *et al.* 2020)
Cell growth promotion	Increase strawberry fruit yield and fruit weight.	*B. amyloliquefaciens* IT45	(Mikiciuk *et al.* 2019)
	Increase percentage of strawberry germination.	*B. amyloliquefaciens* and *Trichoderma harzianum*	(Es-Soufi*et al.* 2020)

**Table 2:** The impact of rhizobacteria on strawberry development and productivity in soilless substrates.

Impact	Species of rhizobacteria	References
Increase root length, stomatal conductance, leaf transpiration, concentration and nutrient CO_2_ content, fertiliser dose.	*Azospirillum brasilense* and *Bacillus uminis*	(da Silva *et al.* 2022)
Increase the biomass, growth, flowering and yield under saline treatment.	*Bacillus methylotrophicus* SMT38, *Bacillus aryabhattai* SMT48 and *Bacillus licheniformis* SMT51	(Redondo-Gómez *et al.* 2022)
Accelerate fruiting time and affected fruit dimensions.	*Pedobacter* sp. CC1	(Morais *et al.* 2019)
Produce more flower, enhance stomatal conductivity and decrease spider mite abundant.	*Azotobacter chroococcum*, *Azospirillum brasilense* and *Pseudomonas brassicacearum*	(Hosseini *et al.* 2022)
Increase the following growth parameters: Plant height, chlorophyll content, fresh and dry root weight.	*Azospirillum* sp. and *Azotobacter* sp.	(Rueda *et al.* 2016)
Increase growth and produce bioactive compound against pathogen *in vitro.*	*Bacillus methylotrophicus*	(Vicente-Hernández *et al.* 2019)
Increase fruit yield and inhibit pathogen growth.	*Bacillus velezensis* IALR619	(Mei *et al.* 2021)

**Table 3:** The impact of rhizobacteria on strawberry quality.

Mechanism	Impact	Rhizobacteria	References
Humic acid and biofertiliser increase nutrient availability	Increase sweetness	Mixed biofertiliser: N_2_-fixing and P-solubilising bacteria	(Hindersah *et al.* 2023)
Vermicompost and biofertiliser increase micronutrients (Mg, Fe, Co)	Increase total soluble solids (TSS)	*Bacillus subtilis, Bacillus megaterium* an*d Pseudomonas uorescen*s	(Kilic *et al.* 2023)
Fulvic acid with biofertiliser as biostimulants	Increase TSS and vitamin C	*Pseudomonas uorescens*	(Martínez-De La Cruz *et al. 2022*)
Rhizobacteria decrease population soil pathogen and increase phosphate availability	Maximize juice pH, increase TSS and total sugar, and decrease acidity	*Pseudomonas orescence, Bacillus subtilis* and *Azotobacter chroococcum*	(Kumar *et al.* 2020)
Rhizobacteria as biocontrol disease	Increase ascorbic acid and anthocyanin content	*Bacillus* sp. RCA3 and *Bacillus* sp. SYB101 or combination with *Pseudomonas* sp. MHA75	(Anuradha *et al.* 2019)
Production IAA phytohormones for improve fruit size and phenolic compounds	Increase fruit length, thickness, total phenolic, content and flavonoid	*Pedobacter* sp.	(Morais *et al.* 2019)
Increase micronutrient uptakes	Increase fruit size, sweetness and flavonoid compounds	*Azospirillum brasilense*	(Pii *et al.* 2018)

**Table 4:** Biocontrol mechanisms of rhizobacteria on the pathogen of strawberry plants.

Pathogen/Diseases	Mechanisms	Species of rhizobacteria	References
*Botrytis cinerea*/Gray mold	Indirect mechanism by induction of systemic resistance.	*Bacillus methylotrophicus*	(Vicente-Hernández *et al.* 2019)
*Botrytis cinerea/*Gray mold	Producing fungal antibiotic compounds.	*Bacillus* sp. and *Pantoea* sp.	(de Moura *et al.* 2021)
*Colletotrichum gloeosporioides* Cg58/*Colletotrichum* crown rot	Antibiotic production.	*Bacillus velezensis* IALR619	(Mei *et al.* 2021)
*Fusarium solani, Rhizoctonia solani* and *Macrophomina phaseolina*/Black rot	*In vitro* test: Antagonistic with unknown mechanisms. In field experiment: Increase peroxidase and chitinase activities in the leaves.	*P. polymyxa* and *B. brevis*	(Abd-El-Kareem *et al.* 2021)
*Botrytis cinerea/*Gray mold	Actinomycin production.	*Streptomyces* sp. sdu1201	(Yong *et al.* 2022)
